# SVA retrotransposons as potential modulators of neuropeptide gene expression

**DOI:** 10.1016/j.npep.2016.09.006

**Published:** 2017-08

**Authors:** Olympia Gianfrancesco, Vivien J. Bubb, John P. Quinn

**Affiliations:** Department of Molecular and Clinical Pharmacology, Institute of Translational Medicine, The University of Liverpool, Liverpool, L69 3BX, UK

**Keywords:** Gene regulation, Retrotransposons, SVA, Evolution, Polymorphism, TRPV1/V3

## Abstract

Many facets of human behaviour are likely to have developed in part due to evolutionary changes in the regulation of neuropeptide and other brain-related genes. This has allowed species-specific expression patterns and unique epigenetic modulation in response to our environment, regulating response not only at the molecular level, but also contributing to differences in behaviour between individuals. As such, genetic variants or epigenetic changes that may alter neuropeptide gene expression are predicted to play a role in behavioural conditions and psychiatric illness. It is therefore of interest to identify regulatory elements that have the potential to drive differential gene expression. Retrotransposons are mobile genetic elements that are known to be drivers of genomic diversity, with the ability to alter expression of nearby genes. In particular, the SINE-VNTR-Alu (SVA) class of retrotransposons is specific to hominids, and its appearance and expansion across the genome has been associated with the evolution of numerous behavioural traits, presumably through their ability to confer unique regulatory properties at the site of their insertion. We review the evidence for SVAs as regulatory elements, exploring how polymorphic variation within these repetitive sequences can drive allele specific gene expression, which would be associated with changes in behaviour and disease risk through the alteration of molecular pathways that are central to healthy brain function.

## Highlights

• SINE-VNTR-Alu (SVA) retrotransposons have been shown to regulate gene expression both *in vitro* and *in vivo*.
• As composite, high GC structures, SVAs may influence gene expression through the modification of local chromatin structure.
• Their polymorphic nature may result in allele-specific expression, contributing to individual differences and disease risk.
• SVAs at TACR3, OXT, and TRPV1/V3 genes suggests a retrotransposon-mediated mechanism contributing to human behaviour.

## 1 Introduction

The movement and insertion of retrotransposable elements across the genome causes mutation that may result in large-scale alterations in gene expression and regulation. With their ability to introduce new splice sites, promoters, and regulatory elements into a locus, active retrotransposition is a driving force for genomic diversity (Cordaux and Batzer, 2009, Erwin et al., 2014), and is likely to have contributed to the species- and tissue-specific expression patterns that we see in primates and humans (Robbez-Masson and Rowe, 2015). While they are often referred to as “jumping genes”, the majority of retrotransposable elements have over time lost their ability to mobilise (Brouha et al., 2003, Mills et al., 2007). However, they still contain many elements that are capable of conferring regulatory properties and may influence the genetic architecture and function at the surrounding loci. In this regard, retrotransposable elements can be considered as genetic elements that may impact the regulation of nearby genes, regardless of any further retrotransposition events. It is in this context that they will herein be considered, and we highlight the importance of these elements in relation to genes of interest discussed by groups at the Aberdeen Neuropeptide Conference 2015, in particular the AVP/Oxytocin locus, as well as the tachykinin receptor TACR3, and voltage gated ion channels TRPV1/V3.

## 2 Overview of transposable elements

Transposable elements (TEs) make up approximately half of the human genome (Lander et al., 2001), and are broadly divided into two categories, named class I and class II, based on their method of replication via a DNA or RNA intermediate. Class II elements include DNA transposons, which make up around 2% of our genome, and are known to have been mobilised as DNA through a cut-and-paste mechanism before losing their ability to mobilise in the human lineage (Bouuaert and Chalmers, 2010, Hancks and Kazazian, 2012).

Alternatively, class I TEs are mobilised and inserted through a two-step process that first involves transcription from the DNA sequence to produce an RNA intermediate. This RNA intermediate is then reverse transcribed into DNA and inserted into a new position in the genome. Class I TEs can be further divided into LTR and non-LTR families, based on the presence or absence of long terminal repeats (LTRs) within the element sequence. LTR retrotransposons include human endogenous retroviruses (HERVs), which make up around 8% of our genome and function in gene regulation, often encoding alternative promoters, resulting in tissue-specific expression (Belshaw et al., 2004).

Non-LTR retrotransposons include Long Interspersed Nuclear Elements (LINEs), Short Interspersed Nuclear Elements (SINEs), and SINE-VNTR-Alus (VNTR = Variable Number Tandem Repeat; SVAs), and are known to be the most abundant class of mobile element in the human genome. While the majority of transposable elements have become inactive throughout the course of evolution, non-LTR retrotransposons are the only group that are known to remain mobile and active within the human lineage (Mills et al., 2007).

SVAs are the most recently evolved family of active non-LTR retrotransposable elements, being present only in the hominid genomes, with approximately 2700–3000 SVA copies in humans (Lander et al., 2001, Savage et al., 2013). These non-autonomous elements contain consensus sequences for LINE-1 endonuclease recognition, and rely directly on active expression of the LINE-1 machinery, ORF1p and ORF2p, in order to be mobilised in *trans* (Hancks et al., 2011, Raiz et al., 2012).

The SVA family of transposable elements can be further divided into 7 subfamilies, named A – F1 in order of age. SVA A is the oldest subfamily in evolutionary terms (~ 13.6 Myrs) and SVA E, F (~ 3.5 and ~ 3.2 Myrs, respectively), and F1 the youngest, whilst subfamilies D and B are the most abundant in the genome, accounting for ~ 40% and ~ 15% of the total number of SVA elements. The youngest subfamilies, SVA E, F, and F1, are human specific, and along with SVA D (~ 9.6 Myrs), are the only subfamilies that remain polymorphic within the human population (Wang et al., 2005).

**Fig. 1:**
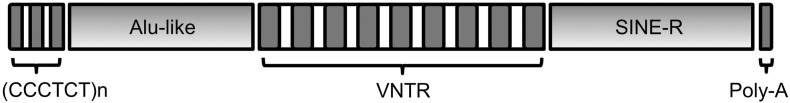
Schematic of a canonical SVA structure showing the five key domains, beginning with a 5′ repeat of the CCCTCT hexamer, followed by an Alu-like structure. The middle of the SVA contains a region of variable number tandem repeats (VNTRs), with older SVAs containing one, and younger SVAs containing two tandem repeat regions. A SINE-R region follows the VNTR, and a poly-A tail marks the 3′ end of the SVA.

Structurally, a canonical SVA is comprised of 5 main components (Fig. 1), beginning with (1) a simple hexamer repeat of (CCCTCT)n at the 5′ end, which may be variable in copy number, followed by (2) an Alu-like region made up of two antisense Alu fragments separated by a region of intervening sequence, (3) one or two variable number tandem repeat (VNTR) regions, typically with a repeating sequence between 35 and 50 bp, (4) a SINE region derived from the 3′ LTR of the retroviral HERV-K10 element, and finally (5) a 3′ poly-A signal (Wang et al., 2005). The seventh SVA family, known as F1, lacks the 5′ CCCTCT hexamer repeat, instead containing a 5′ transduction of exon 1 of the MAST2 gene (Bantysh and Buzdin, 2009).

## 3 SVAs as regulatory elements

Over 60% of SVAs in the human genome are located in genes, or within 10 kb of their flanking sequence (Savage et al., 2013). SVAs are known to be enriched at high GC and gene dense regions, which may suggest preferential insertion in areas such as promoters and actively transcribed regions (Wang et al., 2005). SVAs themselves are generally around 60% GC, with this potentially exceeding 70% within the VNTR region (Wang et al., 2005). The definition of a CpG island is a sequence over 200 bp with a GC content exceeding 50% and an observed-to-expected CpG ratio over 60%. As such, SVAs may be simplistically thought of as mobile CpG islands (Strichman-Almashanu et al., 2002). Indeed, the first exon of MAST2, which has been incorporated into the SVA F1 structure through a 5′ transduction, is defined as a CpG island and has been confirmed to act as a positive transcriptional regulator in human germline cells (Zabolotneva et al., 2012). In this way, SVAs may positively or negatively regulate expression of nearby genes by recruiting transcription factors or altering local chromatin structure depending on epigenetic marks across the element, as has been demonstrated for human endogenous retroviruses (Fasching et al., 2015), causing a region to become either accessible or inaccessible to transcriptional machinery. It is known that retrotransposable elements become hypomethylated in disease states such as cancer (Barchitta et al., 2014, Miousse and Koturbash, 2015), where primate-specific retrotransposons are preferentially affected (Szpakowski et al., 2009), and some retrotransposons become hypomethylated during human ageing (Luo et al., 2014, Jintaridth and Mutirangura, 2010, Bollati et al., 2009). Thus, changes in epigenetic marks across SVAs resulting in, for example, inappropriate reactivation of the element, may contribute to dysregulation of nearby genes and their related pathways, as has been demonstrated for LINE-1 elements in cancer (Hur et al., 2014).

Due to their high GC content, SVAs may also be capable of forming alternative DNA structures which affect transcription, such as G-quadruplexes (G4) (Kejnovsky et al., 2015). G4 structures form in regions containing multiple short runs of G bases, which form planar tetrad structures through Hoogsteen hydrogen bonding and stack together to form helices (Rhodes and Lipps, 2015). Over 40% of human genes contain one or more potential G4 sequences in their promoter region (Huppert and Balasubramanian, 2007), and mutation or stabilisation of the G4 structure has been shown to modify gene expression both *in vitro* and *in vivo* (David et al., 2016, Shin et al., 2015, Gu et al., 2012, Wang et al., 2010)*.* Through sequence analysis, our group have shown that despite accounting for only 0.13% of the total genome, SVAs make up nearly 2% of DNA across the genome that is predicted to form G4 structures. In particular, the 5′ CCCTCT hexamer repeat in SVAs is likely to be the most amenable to G4 formation, and, when accounting for their size, SVAs have the greatest contribution to G4 DNA out of all other genetic elements (Savage et al., 2013). As the evolutionary age of the SVA subtypes decreases, the percentage of sequence capable of quadruplex formation is increased, giving the human-specific SVA E, F, and F1 subtypes the most potential to form transcriptional modulatory G4 structures. This is due in part to the increased copy number of the CCCTCT hexamer repeat in younger SVA subtypes, and also to the increase in VNTR potential to form quadruplex, as the younger subtypes contain two VNTRs with a higher GC content, as opposed to one with lower GC content in the older SVAs. For example, the SVA F1 subtype lacks the 5′ hexamer, and thus its G4 potential is conferred entirely by the high GC VNTR regions (Savage et al., 2013).

We have demonstrated the regulatory properties of both full length SVAs as well as their individual components using reporter gene constructs both *in vitro* and *in vivo*, in cell lines and chick embryo models (Savage et al., 2013, Savage et al., 2014). For example, the Parkinson's related gene, PARK7, has a full length human-specific SVA D located approximately 8 kb from the major transcriptional start site. Reporter gene experiments showed that the PARK7 SVA can positively modulate expression *in vitro*, with a truncation lacking the SINE domain showing the greatest enhancer capability. Individually, the tandem repeat region of this SVA acted as a negative regulator of transcription, identifying a range of modulatory effects conferred by the distinct elements in this composite structure (Savage et al., 2013). Similarly, our analysis of the primate-specific SVA D, which lies 10 kb upstream of the FUS gene, verified the regulatory properties of this SVA *in vivo* using a chick embryo model. Again, different components of the SVA displayed different regulatory properties, with the central VNTR region acting as a positive transcriptional regulator, whereas the complete SVA acted to decrease reporter gene expression. GFP imaging studies in the chick embryo revealed activity of the FUS SVA in the developing neural tube (Savage et al., 2014).

Kim et al. have also demonstrated the capacity of SVAs to act as novel promoters through bioinformatic analysis of human-specific insertions that are expressed as exons in the transcriptome. Although this analysis would not capture SVAs acting as more distant regulators, Kim et al. identified 12 cases in which human-specific SVA insertions led to the formation of novel promoters, driving expression of human-specific transcripts originating from within the SVA (Kim and Hahn, 2011). For example, an SVA insertion 5′ to the TBPL2 gene has been shown to act as a novel promoter for a human-specific transcript of TBPL2, in which SVA derived sequence is included as a novel first exon. Similarly, an SVA insertion at intron 19 of the WDR66 gene resulted in expression of a human-specific truncated isoform of WDR66, containing exons 20–22 (Kim and Hahn, 2011). SVA insertions have also been shown to drive expression of novel antisense transcripts, including a human-specific SVA insertion at intron 45 of the SYNE2 gene, which acts as a novel promoter for the expression of six antisense ESTs (Kim and Hahn, 2010).

Multiple components of the SVA composite structure are known to regulate neuropeptide gene expression individually. VNTRs, which are a major component of SVAs, have been demonstrated to be key neuropeptide regulatory domains. For example, the arginine vasopressin 1a receptor (AVPR1A) contains two upstream VNTRs named RS1 and RS3. These repetitive regions were originally identified in the prairie vole, and polymorphisms were found to be associated with monogamous and non-monogamous behaviour between different species (Hammock et al., 2005, Hammock and Young, 2002). Tansey et al. have demonstrated the allele-specific regulatory effects of both repetitive regions, with the long alleles of both the RS1 and RS3 supporting increased transcription of a reporter gene over the shorter alleles in the SH-SY5Y neuroblastoma cell line (Tansey et al., 2011). Further studies have shown that differences in tandem repeat number at the promoter of AVPR1A contributes to differences in tissue-specific expression, resulting in species-specific receptor distribution across the brain and contributing to behavioural diversity (Donaldson and Young, 2013). In humans, variation at the AVPR1A RS1 and RS3 has been associated with differences in behavioural traits such as pair bonding (Walum et al., 2008). Variation at these regions is known to interact with environmental factors such as childhood adversity to mediate social behaviour in adulthood (Liu et al., 2015), and has been shown to correlate with autism spectrum behaviour in Finnish and Korean populations (Kantojarvi et al., 2015, Yang et al., 2010).

Similarly, expression of the POMC gene in both the human and mouse hypothalamus is co-ordinated by the action of two distal regulatory elements, named nPE1 and nPE2, which act as neuronal enhancers (de Souza et al., 2005). By comparing genomes from multiple species, Santangelo et al. demonstrated that nPE2 originated from a SINE retrotransposon insertion at this locus in the evolutionary lineage leading to mammals, thereby conferring distinct regulatory properties to the mammalian POMC gene in hypothalamic neurons (Santangelo et al., 2007). Kuehnen et al. have also demonstrated the regulatory effects of Alu element insertion into the POMC gene through evolution. The human intron 2 of POMC contains three Alu insertions and the CpG island at this region is hypermethylated. A similar pattern of hypermethylation at this region is seen in chimpanzees, who also have Alu insertions at the POMC intron 2. However, in the mouse and in more distant primates such as lemurs, no Alu insertions are present in POMC intron 2 and consequently little to no methylation is found across this region. This suggests that the insertion of retrotransposons into this neuropeptide gene through evolution has resulted in species-specific methylation patterns which are likely to result in altered expression of POMC in higher primates (Kuehnen et al., 2012).

**Fig. 2:**
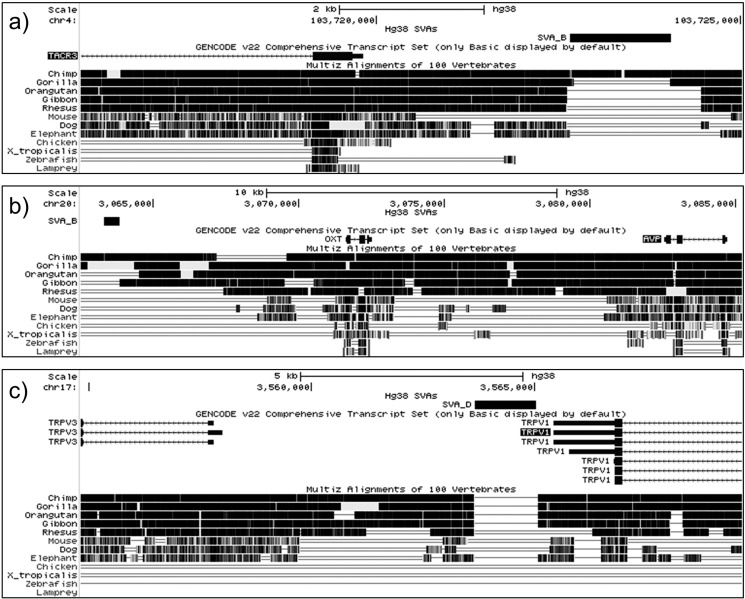
Schematic of the TACR3, OXT, and TRPV1/V3 genes, displaying the positions and evolutionary conservation of SVAs at these loci. (a) The TACR3 gene has a human- and chimp-specific SVA B beginning less than 3 kb upstream of the transcriptional start site. (b) Similarly, a human- and chimp- specific SVA B lies less than 8 kb upstream of the OXT transcriptional start site. (c) Finally, a human-specific SVA D is present within the intervening 8 kb of sequence separating the TRPV1 and TRPV3 genes, with the SVA residing approximately 6 kb upstream of TRPV3 and directly adjacent to the 3′ end of TRPV1. We hypothesise that insertion of SVAs at these loci may have altered regulation of neuropeptide related gene expression during hominid evolution.

As SVA are composite structures which include SINE, VNTR and Alu sequences, their presence in genic regions may influence transcription in a manner similar to the above. Observation of neuropeptide-related gene loci revealed SVA insertions in the upstream regions of the tachykinin TACR3 receptor (Fig. 2a), the oxytocin encoding OXT (Fig. 2b), and between the cation channel TRPV1 and TRPV3 genes (Fig. 2c), with the latter having an extensive literature on modulation of neuropeptide function and release. Both TACR3 and OXT have a chimp- and human-specific SVA B beginning less than 3 kb and 8 kb upstream of their respective promoters, which may have conferred distinct regulation of tachykinin receptor 3 and oxytocin in humans and chimpanzees. Vanilloid receptors such as TRPV1 and TRPV3 are also of interest in understanding neuropeptide function, as their activation leads to release of substance P, therefore demonstrating a close relationship between the two systems in many areas of neuronal signalling (Keszthelyi et al., 2013). The TRPV1/V3 genes are separated by approximately 8 kb between the 5′ end of TRPV3 and the 3′ end of TRPV1, and this region contains a human-specific SVA D. The SVA at this locus is less than 6 kb upstream of the TRPV3 transcriptional start site, and immediately adjacent to the end of TRPV1, thus allowing it to potentially modulate the expression of both genes. In a similar manner, the SVA upstream of the OXT gene could potentially regulate the nearby AVP gene at this locus. We hypothesise that these hominid-specific retrotransposons would therefore impart specific regulatory properties upon these genes that may allow for evolutionary changes in their tissue-specific or stimulus inducible regulation.

## 4 The polymorphic nature of SVAs

SVAs are considered to be the most polymorphic elements in the human genome, not just in structure but also for their presence or absence (Bennett et al., 2004). Although mobile genetic elements are often stringently repressed to prevent deleterious changes to the cell's genome through further mobilisation and insertion (Yang and Wang, 2016), active retrotransposition is known to occur during normal differentiation of neuronal stem cells, and in adult neurogenesis (Coufal et al., 2009, Muotri et al., 2009). As such, many of our brain cells contain unique retrotransposon insertions. Although the exact rate is currently an area of controversy (Upton et al., 2015, Hazen et al., 2016, Evrony et al., 2016), all groups agree that active mobilisation of retrotransposons occurs in the brain. Higher estimates suggest that each hippocampal neuron contains approximately 13.7 new somatic LINE-1 insertions on average, which are highly enriched at brain-related gene loci (Upton et al., 2015), however differences in the estimated rates may result from differing sensitivities of the techniques used, and may also differ by cell type.

The SVA structure itself is composed of multiple repetitive domains, each of which may contribute to polymorphic variation in the SVA due to differences in copy number. Within the aforementioned PARK7 SVA, we identified three common variants of the CCCTCT hexamer VNTR, with 7, 10, or 13 copies of the repeat. Similarly, we have shown variation in length in the terminal poly-A stretch in other retrotransposable elements (Payton et al., 2016). However, the most likely region for copy number variation is the large central VNTR domain, which we have shown to be polymorphic in both the PARK7 SVA and the FUS SVA. We identified three distinct alleles of the central VNTR in the PARK7 SVA and two alleles in the FUS SVA (Savage et al., 2013, Savage et al., 2014).

## 5 Summary

Overall, our work suggests that SVA elements can alter gene expression through their capacity for local transcriptional and epigenetic modulation. We hypothesise that SVA insertions at the OXT/AVP, TACR3, and TRPV1/V3 loci, all genes discussed at the Aberdeen Neuropeptide Conference 2015, have contributed to the evolution of species-specific regulation and expression of these genes in humans and other primates, allowing the evolution of higher cognitive and social functioning, as well as variation at these loci acting in combination with our environment to modulate human behaviour. Their high GC content may allow them to act through a similar mechanism to CpG islands, altering the transcriptional availability of a locus depending on epigenetic markers across the element, as demonstrated for the Alu retrotransposon in the POMC gene. Similarly, their high G content means that SVAs have the potential to form alternative DNA structures such as G-quadruplex, which is known to alter transcription and facilitate enhancer-promoter interactions to regulate gene expression across a locus. Variation within SVA elements also provides an additional mechanism to drive differential gene expression through allele-specific transcription factor binding and identifies them as potential biomarkers for disease. The presence of SVAs at neuropeptide gene loci points to a retrotransposon-mediated evolutionary mechanism which may have contributed to the development of human behavioural traits.

## References

[bb0005] Bantysh O.B., Buzdin A.A. (2009). Novel family of human transposable elements formed due to fusion of the first exon of gene MAST2 with retrotransposon SVA. Biochemistry (Mosc).

[bb0010] Barchitta M., Quattrocchi A., Maugeri A., Vinciguerra M., Agodi A. (2014). LINE-1 hypomethylation in blood and tissue samples as an epigenetic marker for cancer risk: a systematic review and meta-analysis. PLoS One.

[bb0015] Belshaw R., Pereira V., Katzourakis A., Talbot G., Paces J., Burt A., Tristem M. (2004). Long-term reinfection of the human genome by endogenous retroviruses. Proc. Natl. Acad. Sci. U. S. A..

[bb0020] Bennett E.A., Coleman I.E., Tsui C., Pittard W.S., Devine S.E. (2004). Natural genetic variation caused by transposable elements in humans. Genetics.

[bb0025] Bollati V., Schwartz J., Wright R., Litonjua A., Tarantini L., Suh H., Sparrow D., Vokonas P., Baccarelli A. (2009). Decline in genomic DNA methylation through aging in a cohort of elderly subjects. Mech. Ageing Dev..

[bb0030] Brouha B., Schustak J., Badge R.M., Lutz-Prigge S., Farley A.H., Moran J.V., Kazazian H.H. (2003). Hot L1s account for the bulk of retrotransposition in the human population. Proc. Natl. Acad. Sci. U. S. A..

[bb0035] Bouuaert C.C., Chalmers R. (2010). Transposition of the human Hsmar1 transposon: rate-limiting steps and the importance of the flanking TA dinucleotide in second strand cleavage. Nucleic Acids Res..

[bb0040] Cordaux R., Batzer M.A. (2009). The impact of retrotransposons on human genome evolution. Nat. Rev. Genet..

[bb0045] Coufal N.G., Garcia-Perez J.L., Peng G.E., Yeo G.W., Mu Y., Lovci M.T., Morell M., O'shea K.S., Moran J.V., Gage F.H. (2009). L1 retrotransposition in human neural progenitor cells. Nature.

[bb0050] David A.P., Margarit E., Domizi P., Banchio C., Armas P., Calcaterra N.B. (2016). G-quadruplexes as novel cis-elements controlling transcription during embryonic development. Nucleic Acids Res..

[bb0055] De Souza F.S., Santangelo A.M., Bumaschny V., Avale M.E., Smart J.L., Low M.J., Rubinstein M. (2005). Identification of neuronal enhancers of the proopiomelanocortin gene by transgenic mouse analysis and phylogenetic footprinting. Mol. Cell. Biol..

[bb0060] Donaldson Z.R., Young L.J. (2013). The relative contribution of proximal 5′ flanking sequence and microsatellite variation on brain vasopressin 1a receptor (Avpr1a) gene expression and behavior. PLoS Genet..

[bb0065] Erwin J.A., Marchetto M.C., Gage F.H. (2014). Mobile DNA elements in the generation of diversity and complexity in the brain. Nat. Rev. Neurosci..

[bb0070] Evrony G.D., Lee E., Park P.J., Walsh C.A. (2016). Resolving rates of mutation in the brain using single-neuron genomics. Elife.

[bb0075] Fasching L., Kapopoulou A., Sachdeva R., Petri R., Jonsson M.E., Manne C., Turelli P., Jern P., Cammas F., Trono D., Jakobsson J. (2015). TRIM28 represses transcription of endogenous retroviruses in neural progenitor cells. Cell Rep..

[bb0080] Gu H.P., Lin S., Xu M., Yu H.Y., Gu X.J., Zhang Y.Y., Yuan G., Gao W. (2012). Up-regulating relaxin expression by G-quadruplex interactive ligand to achieve antifibrotic action. Endocrinology.

[bb0085] Hammock E.A., Young L.J. (2002). Variation in the vasopressin V1a receptor promoter and expression: implications for inter- and intraspecific variation in social behaviour. Eur. J. Neurosci..

[bb0090] Hammock E.A., Lim M.M., Nair H.P., Young L.J. (2005). Association of vasopressin 1a receptor levels with a regulatory microsatellite and behavior. Genes Brain Behav..

[bb0095] Hancks D.C., Kazazian H.H. (2012). Active human retrotransposons: variation and disease. Curr. Opin. Genet. Dev..

[bb0100] Hancks D.C., Goodier J.L., Mandal P.K., Cheung L.E., Kazazian H.H. (2011). Retrotransposition of marked SVA elements by human L1s in cultured cells. Hum. Mol. Genet..

[bb0105] Hazen J.L., Faust G.G., Rodriguez A.R., Ferguson W.C., Shumilina S., Clark R.A., Boland M.J., Martin G., Chubukov P., Tsunemoto R.K., Torkamani A., Kupriyanov S., Hall I.M., Baldwin K.K. (2016). The complete genome sequences, unique mutational spectra, and developmental potency of adult neurons revealed by cloning. Neuron.

[bb0110] Huppert J.L., Balasubramanian S. (2007). G-quadruplexes in promoters throughout the human genome. Nucleic Acids Res..

[bb0115] Hur K., Cejas P., Feliu J., Moreno-Rubio J., Burgos E., Boland C.R., Goel A. (2014). Hypomethylation of long interspersed nuclear element-1 (LINE-1) leads to activation of proto-oncogenes in human colorectal cancer metastasis. Gut.

[bb0120] Jintaridth P., Mutirangura A. (2010). Distinctive patterns of age-dependent hypomethylation in interspersed repetitive sequences. Physiol. Genomics.

[bb0125] Kantojarvi K., Oikkonen J., Kotala I., Kallela J., Vanhala R., Onkamo P., Jarvela I. (2015). Association and promoter analysis of AVPR1A in Finnish autism families. Autism Res..

[bb0130] Kejnovsky E., Tokan V., Lexa M. (2015). Transposable elements and G-quadruplexes. Chromosom. Res..

[bb0135] Keszthelyi D., Troost F.J., Jonkers D.M., Helyes Z., Hamer H.M., Ludidi S., Vanhoutvin S., Venema K., Dekker J., Szolcsanyi J., Masclee A.A. (2013). Alterations in mucosal neuropeptides in patients with irritable bowel syndrome and ulcerative colitis in remission: a role in pain symptom generation?. Eur. J. Pain.

[bb0140] Kim D.S., Hahn Y. (2010). Human-specific antisense transcripts induced by the insertion of transposable element. Int. J. Mol. Med..

[bb0145] Kim D.S., Hahn Y. (2011). Identification of human-specific transcript variants induced by DNA insertions in the human genome. Bioinformatics.

[bb0150] Kuehnen P., Mischke M., Wiegand S., Sers C., Horsthemke B., Lau S., Keil T., Lee Y.A., Grueters A., Krude H. (2012). An Alu element-associated hypermethylation variant of the POMC gene is associated with childhood obesity. PLoS Genet..

[bb0155] Lander E.S., Linton L.M., Birren B., Nusbaum C., Zody M.C., Baldwin J., Devon K., Dewar K., Doyle M., Fitzhugh W., Funke R., Gage D., Harris K., Heaford A., Howland J., Kann L., Lehoczky J., Levine R., Mcewan P., Mckernan K., Meldrim J., Mesirov J.P., Miranda C., Morris W., Naylor J., Raymond C., Rosetti M., Santos R., Sheridan A., Sougnez C., Stange-Thomann Y., Stojanovic N., Subramanian A., Wyman D., Rogers J., Sulston J., Ainscough R., Beck S., Bentley D., Burton J., Clee C., Carter N., Coulson A., Deadman R., Deloukas P., Dunham A., Dunham I., Durbin R., French L., Grafham D., Gregory S., Hubbard T., Humphray S., Hunt A., Jones M., Lloyd C., Mcmurray A., Matthews L., Mercer S., Milne S., Mullikin J.C., Mungall A., Plumb R., Ross M., Shownkeen R., Sims S., Waterston R.H., Wilson R.K., Hillier L.W., Mcpherson J.D., Marra M.A., Mardis E.R., Fulton L.A., Chinwalla A.T., Pepin K.H., Gish W.R., Chissoe S.L., Wendl M.C., Delehaunty K.D., Miner T.L., Delehaunty A., Kramer J.B., Cook L.L., Fulton R.S., Johnson D.L., Minx P.J., Clifton S.W., Hawkins T., Branscomb E., Predki P., Richardson P., Wenning S., Slezak T., Doggett N., Cheng J.F., Olsen A., Lucas S., Elkin C., Uberbacher E., Frazier M. (2001). Initial sequencing and analysis of the human genome. Nature.

[bb0160] Liu J.J., Lou F., Lavebratt C., Forsell Y. (2015). Impact of childhood adversity and vasopressin receptor 1a variation on social interaction in adulthood: a cross-sectional study. PLoS One.

[bb0165] Luo Y., Lu X., Xie H. (2014). Dynamic Alu methylation during normal development, aging, and tumorigenesis. Biomed. Res. Int..

[bb0170] Mills R.E., Bennett E.A., Iskow R.C., Devine S.E. (2007). Which transposable elements are active in the human genome?. Trends Genet..

[bb0175] Miousse I.R., Koturbash I. (2015). The fine LINE: methylation drawing the cancer landscape. Biomed. Res. Int..

[bb0180] Muotri A.R., Zhao C., Marchetto M.C., Gage F.H. (2009). Environmental influence on L1 retrotransposons in the adult hippocampus. Hippocampus.

[bb0185] Payton A., Sindrewicz P., Pessoa V., Platt H., Horan M., Ollier W., Bubb V.J., Pendleton N., Quinn J.P. (2016). A TOMM40 poly-T variant modulates gene expression and is associated with vocabulary ability and decline in nonpathologic aging. Neurobiol. Aging.

[bb0190] Raiz J., Damert A., Chira S., Held U., Klawitter S., Hamdorf M., Lower J., Stratling W.H., Lower R., Schumann G.G. (2012). The non-autonomous retrotransposon SVA is trans-mobilized by the human LINE-1 protein machinery. Nucleic Acids Res..

[bb0195] Rhodes D., Lipps H.J. (2015). G-quadruplexes and their regulatory roles in biology. Nucleic Acids Res..

[bb0200] Robbez-Masson L., Rowe H.M. (2015). Retrotransposons shape species-specific embryonic stem cell gene expression. Retrovirology.

[bb0205] Santangelo A.M., De Souza F.S., Franchini L.F., Bumaschny V.F., Low M.J., Rubinstein M. (2007). Ancient exaptation of a CORE-SINE retroposon into a highly conserved mammalian neuronal enhancer of the proopiomelanocortin gene. PLoS Genet..

[bb0210] Savage A.L., Bubb V.J., Breen G., Quinn J.P. (2013). Characterisation of the potential function of SVA retrotransposons to modulate gene expression patterns. BMC Evol. Biol..

[bb0215] Savage A.L., Wilm T.P., Khursheed K., Shatunov A., Morrison K.E., Shaw P.J., Shaw C.E., Smith B., Breen G., Al-Chalabi A., Moss D., Bubb V.J., Quinn J.P. (2014). An evaluation of a SVA retrotransposon in the FUS promoter as a transcriptional regulator and its association to ALS. PLoS One.

[bb0220] Shin Y.J., Kumarasamy V., Camacho D., Sun D. (2015). Involvement of G-quadruplex structures in regulation of human RET gene expression by small molecules in human medullary thyroid carcinoma TT cells. Oncogene.

[bb0225] Strichman-Almashanu L.Z., Lee R.S., Onyango P.O., Perlman E., Flam F., Frieman M.B., Feinberg A.P. (2002). A genome-wide screen for normally methylated human CpG islands that can identify novel imprinted genes. Genome Res..

[bb0230] Szpakowski S., Sun X., Lage J.M., Dyer A., Rubinstein J., Kowalski D., Sasaki C., Costa J., Lizardi P.M. (2009). Loss of epigenetic silencing in tumors preferentially affects primate-specific retroelements. Gene.

[bb0235] Tansey K.E., Hill M.J., Cochrane L.E., Gill M., Anney R.J., Gallagher L. (2011). Functionality of promoter microsatellites of arginine vasopressin receptor 1A (AVPR1A): implications for autism. Mol. Autism..

[bb0240] Upton K.R., Gerhardt D.J., Jesuadian J.S., Richardson S.R., Sanchez-Luque F.J., Bodea G.O., Ewing A.D., Salvador-Palomeque C., Van Der Knaap M.S., Brennan P.M., Vanderver A., Faulkner G.J. (2015). Ubiquitous L1 mosaicism in hippocampal neurons. Cell.

[bb0245] Walum H., Westberg L., Henningsson S., Neiderhiser J.M., Reiss D., Igl W., Ganiban J.M., Spotts E.L., Pedersen N.L., Eriksson E., Lichtenstein P. (2008). Genetic variation in the vasopressin receptor 1a gene (AVPR1A) associates with pair-bonding behavior in humans. Proc. Natl. Acad. Sci. U. S. A..

[bb0250] Wang H., Xing J., Grover D., Hedges D.J., Han K., Walker J.A., Batzer M.A. (2005). SVA elements: a hominid-specific retroposon family. J. Mol. Biol..

[bb0255] Wang X.D., Ou T.M., Lu Y.J., Li Z., Xu Z., Xi C., Tan J.H., Huang S.L., An L.K., Li D., Gu L.Q., Huang Z.S. (2010). Turning off transcription of the bcl-2 gene by stabilizing the bcl-2 promoter quadruplex with quindoline derivatives. J. Med. Chem..

[bb0260] Yang F., Wang P.J. (2016). Multiple LINEs of retrotransposon silencing mechanisms in the mammalian germline. Semin. Cell Dev. Biol..

[bb0265] Yang S.Y., Cho S.C., Yoo H.J., Cho I.H., Park M., Yoe J., Kim S.A. (2010). Family-based association study of microsatellites in the 5′ flanking region of AVPR1A with autism spectrum disorder in the Korean population. Psychiatry Res..

[bb0270] Zabolotneva A.A., Bantysh O., Suntsova M.V., Efimova N., Malakhova G.V., Schumann G.G., Gayfullin N.M., Buzdin A.A. (2012). Transcriptional regulation of human-specific SVAF(1) retrotransposons by cis-regulatory MAST2 sequences. Gene.

